# Nature conservation versus climate protection: a basic conflict of goals regarding the acceptance of climate protection measures?

**DOI:** 10.3389/fpsyg.2023.1114677

**Published:** 2023-06-26

**Authors:** Anke Blöbaum, Lukas Engel, Katrin Beer, Michael Böcher, Ellen Matthies

**Affiliations:** ^1^Faculty of Natural Sciences, Institute of Psychology, Environmental Psychology, Otto-von-Guericke-University Magdeburg, Magdeburg, Germany; ^2^Chair for Political Science and Sustainable Development, Institute of Political Science, Faculty of Humanities, Social Science and Education, Otto-von-Guericke University Magdeburg, Magdeburg, Germany; ^3^Chair of Environmental and Climate Policy, Department of Science, Technology and Society/Bavarian School of Public Policy, Technical University of Munich, Munich, Germany

**Keywords:** nature conservation, climate protection, conflict of goals, biospheric value orientation, personal norm, political orientation, global human identity

## Abstract

Transformation processes are embedded in a broader discourse on sustainability, climate protection, and biodiversity protection. In this context, possible interindividual conflicts between an interest in nature conservation and efforts to counteract climate change also seem to be relevant. This study focuses on the acceptability of different climate protection measures with possible impacts on landscapes, habitats, and human recreation. Based on a survey of a representative sample (*N* = 1,427 participants), the impact of conservation-related beliefs on the acceptance of four different climate protection measures was analyzed with respect to possible conflicts with values and norms relevant to climate protection. The study focuses in particular on potential value-based conflicts, as this type of conflict is classified as non-negotiable in negotiation processes and thus represents a particular social challenge. Also, to consider the possible relevance of political orientation and humanity orientation, eight structural equation models were tested. Results showed similar structures for the acceptance of the four climate protection measures. There did not seem to be value-based conflicts between nature conservation and climate protection, as the results showed substantial similarities between nature conservation beliefs grounded in biospheric value orientation (protecting biodiversity) and values and norms relevant for climate protection. Political orientation seemed to be relevant as well, as left-oriented people were more likely to accept the four climate protection measures that were tested. However, the relationship between political orientation and acceptance of the measures was – without exception – mediated by the personal norm.

## 1. Introduction

Climate change and the associated urgent need for climate protection measures have become central challenges that our society faces. The Intergovernmental Panel on Climate Change (IPCC) has made it clear that the goal of the Paris Agreement to limit global warming to 1.5 degrees is still possible but requires immediate action and appropriate climate policy measures (IPCC, 2022). In this context, the shift toward a sustainable and climate-friendly economic system (D’Amato et al., 2017; Jaeger-Erben et al., 2021; Thrän and Moesenfechtel, 2022) requires the use of renewable energy and renewable resources for material use.

The development and implementation of renewable energy technologies (e.g., wind turbines, photovoltaic systems, and wood energy plants), the transformations of energy systems (e.g., electrification, decarbonization, defossilization, biologization, and decentralization), and associated climate and energy policies (e.g., Paris Agreement; United Nations Framework Convention on Climate Change; Renewable Energy Directive, European Union; Renewable Energy Act, Germany) have already been intensively researched and discussed – especially for the power sector (Jacobsson and Lauber, 2006; Rogelj et al., 2015; Bush, 2020; Haji Esmaeili et al., 2020; Malladi and Sowlati, 2020; Nian et al., 2022).

The expansion of renewables and the promotion and use of other sensible technologies to mitigate global warming can succeed only if the citizens support the associated political measures that support such technologies and their implementation. In particular, the acceptance of the construction and repowering of wind power plants has already been intensively investigated in this area (e.g., Devine-Wright, 2005; Wüstenhagen et al., 2007; Ek and Matti, 2015; Petrova, 2016).

In this context, it is first necessary to precisely define the concept of acceptance. Wüstenhagen et al. (2007) distinguished three levels of acceptance, namely, *community acceptance, socio-political acceptance,* and *market acceptance*. While community acceptance refers to the project level, and focuses on a specific, local plant project, socio-political acceptance refers to the social acceptance of the technology on a more general level, and market acceptance describes the process by which the market adopts the technological innovation (Wüstenhagen et al., 2007).

This paper focuses on the socio-political acceptance of selected climate policy measures and examines relevant predictors. Although acceptance of or concerns about local projects or specific plants are not considered in the following, we assume that the possible predictors analyzed in this study (values, beliefs, norms, political orientation, and global human identity; see below) might also be relevant for understanding community acceptance. Nevertheless, the local context will play a major role and must be considered in these cases as well, and research focusing on community acceptance, has to take into account the specific situational factors as well as the perception of procedural justice (Tyler, 2000) and/or distributional justice (Adams, 1965).

While the socio-political acceptance of the extension of renewable energies with the aim of climate protection is relatively high in Germany, for example^1^ (BfN (Bundesamt für Naturschutz), 2022), there are definitely local protests, especially with regard to the extension of onshore wind power plants (Fachagentur Windenergie (FA Wind), 2021). The reasons for these protests are mainly protests against intrusions that change the landscape, harm biodiversity by destroying sensitive habitats, but also the potential that local residents will be annoyed by the anticipated noise of wind power or by wind turbine obstruction lights [Fachagentur Windenergie (FA Wind), 2019; Quentin, 2019; Pohl et al., 2021; Gaßner et al., 2022; Kopernikus-Projekt Ariadne, 2022].

In addition to the choice of renewable energy technologies and certain locations (van der Horst, 2007), several other factors can have a critical influence on both the socio-political acceptance of technologies and measures and the overall success of the transition process: the actual design of the transformation process (participation, procedural and distributive justice; Heleno et al., 2022; Reitz et al., 2022), the characteristics and perceptions of a problem (Wolsink, 2007; Beer, 2021), and the choice of policy instruments (Böcher, 2012; Barnea et al., 2022; Berker and Böcher, 2022). Social opposition against renewable energy – concerning local plants or renewable energy technologies and climate protection measures in general – or other innovative technologies can be based on different motives, different values, and conflicting goals (Baasch, 2021; Baasch et al., 2021; Berntsen et al., 2021; Sovacool et al., 2022; Zawadzki et al., 2022).

Protests based on local residents’ *annoyance* with renewable energy plants are (mainly) directly linked to the characteristics of these specific plants and can therefore be understood as particular conflicts of interest between specific groups from the population (residents) and the planned extension of renewable energies. Protests based on fears of endangering habitats and impairing biodiversity, on the other hand, could also refer to a fundamental conflict in goals between nature conservation goals and climate protection goals. For example, wind turbines might pose a threat to birds and bats, and the construction of solar panels could take up land that may be lost for conservation purposes. These goal conflicts could also be expected for other relevant climate protection technologies that are also associated with landscape interventions and impairment of habitats, such as technologies associated with forest management for carbon benefits such as wood for the building sector (Eisele and Juschka, 2022; proHolz Bayern, 2022). Does this expectation imply that there is a fundamental conflict of interest between nature conservation and climate protection and that nature conservationists and climate protectionists are opposing each other?

We do not want to suggest that the goals of nature conservation and climate protection are fundamentally contradictory. For example, a joint publication by the Intergovernmental Panel on Climate Change (IPCC) and the Intergovernmental Science-Policy Platform on Biodiversity and Ecosystem Services (IPBES; Pörtner et al., 2021) argues for a close causal link between climate protection and nature conservation. Even though it appears plausible that the conservation of biodiversity and habitats depends on the mitigation of climate change, the conflict between climate protection and nature conservation goals seems to be a socio-political problem, at least in Germany. Possible conflicts of goals here might partly be due to the fact that from the nature conservation perspective, climate protection is promoted at the “expense of nature,” i.e., nature is not primarily seen as a *victim* of climate change, but as a *resource* for solving the problem (BUND, 2009; Carstens, 2013; Schultz, 2020). In addition, there are indications of right-wing political interventions in nature conservation that instrumentalize nature conservation motives politically in order to intensify a conflict of goals between nature conservation and climate protection and thus also suggest an underlying conflict of values [for a critical overview see FARN (Fachstelle Radikalisierungsprävention und Engagement im Naturschutz), 2019; Gottschlich and Katz, 2020; NABU (Naturschutzbund Deutschland), 2022].

### 1.1. Types of conflicts between nature conservation motives and climate protection motives

We would like to start with a brief description of different causes of disputes or possible types of interindividual conflicts between nature conservation motives and climate protection motives. Moore (2014) proposed the subdivision of (1) conflicts of interest, (2) relationship-based conflicts, (3) value-based conflicts, and (4) structural conflicts. In real life situations, there are quite often mixed forms of types of conflicts, or several types of conflicts occurring at the same time. It is nevertheless important to make these distinctions because different types of conflicts have very different consequences for possible conflict management and negotiation.

In the following we will not deal more intensively with relationship-based conflicts and structural conflicts, because relationship-based conflicts can occur in all areas of social life, while the management of structural conflicts requires information about the respective situation-specific and local parameters. So let us take a closer look at possible conflicts of interest and possible value-based conflicts. Conflicts of interest are always embedded in a more or less complex, specific context, i.e., involved groups or individuals pursue different goals in a specific situation, or at least believe that goals are incompatible. As long as there are no underlying conflicting values, these goals are basically negotiable. An appropriate solution strategy for a real conflict of interest would be to convert positions into interests in order to negotiate compromises or compensations. Such a conflict of interest could exist, for example, if residents feel annoyed by the noise or obstruction lights from wind turbines.

The picture is different in the case of value-based conflicts, that pose a particular challenge for conflict mediation: Values are basically considered to be difficult, if not impossible, to negotiate (Illes et al., 2014; Stöckli and Tanner, 2014). In these cases, it is sometimes reasonable only to examine the extent to which it is at least possible to develop overarching common goals.

For example, if conservation-related reasons are cited against the expansion of specific renewable energy plants or forestry technologies, it is important to understand whether these are conflicts of interest that are *fundamentally negotiable* or whether there is an underlying conflict of values that may inhibit the identification of common overarching goals and thus hinder the resolution of the conflict.

This may illustrate the importance of analyzing whether societal disputes about climate and/or nature protection measures are based on value-based conflicts or whether these can rather be neglected and thus the opportunity for negotiation is given.

Before we try to shed light on this question empirically, we first take a closer look at relevant conservation-related values and beliefs and deduce the extent to which these might come into conflict with values and beliefs that are relevant to climate protection. We complement our perspective by considering political orientation and global human identity as other possible contributing factors in this context.

### 1.2. Relevance of values, beliefs, and norms for nature conservation or climate protection

In the following, we discuss possible relevant values/value orientations and beliefs as predictors of environmental protection/climate protection and nature conservation. We do not claim to present an entire list of relevant factors that (might) have an impact on pro-environmental behavior and/or conservation behavior, but deliberately focus on values and norms. As already mentioned, we consider it particularly important to look for *value-based conflicts* in the domain of socio-political acceptance of climate and nature protection measures. Approaches that rely mainly on rational choice models and thus can explain environmental behavior quite successfully, such as approaches in the tradition of the Theory of Planned Behavior (TPB, Ajzen, 1991), are therefore excluded from the following description.

The distinction of environmental protection/climate protection on the one hand side and nature conservation on the other hand side is of primary importance for this paper, so we have deliberately limited ourselves to refer to studies that explicitly separate nature and the environment or nature conservation and environmental/climate protection. We will start with the area of nature conservation and then move on to climate protection.

There is already a longer social-empirical research tradition dealing with approaches for explaining conservation motives. Among others, the egocentric-anthropocentric dimension has been used to explain the relevance of values (e.g., Stern and Dietz, 1994). However, the suggestion of an “egocentric-anthropocentric” dimension seems to be an oversimplification of the rather complex structure of beliefs and values regarding nature and conservation (Buijs et al., 2006; Buijs, 2009). Nature-related beliefs seem to be more associated with the (cognitive) image of nature, e.g., concepts about the relationship between nature and culture or the fragility of nature (Fischer and van der Wal, 2007; Buijs, 2009), whereas nature-related values reflect the normative dimension. Because we are interested primarily in possible conflicts between nature conservation and climate protection in this paper, we mainly refer to this normative dimension in the following.

According to Rockeach (1973), values can be understood in terms of standards that serve as guiding principles in a person’s life, are stable over time, and are rather general and independent of concrete situations. Stern et al. (1995) offered a link between basic values (Schwartz, 1992) and environmental value orientations. They took items from Schwartz’s value instrument to reflect egoistic (at the level of one’s own person), social-altruistic (at the level of other people), and biospheric (at the level of non-human life) value orientations. More recent studies have also taken into account the significance of value orientations for the context of nature conservation (Martin and Czellar, 2017; Fornara et al., 2020; Molinario et al., 2020). Fornara et al. (2020) analyzed committed action for nature and biodiversity based on an extended version of the value-belief-norm theory in 7 European countries and were able to demonstrate a direct influence of biospheric values not only on norms but also directly on action toward biodiversity. Martin and Czellar (2017) focused on biospheric values as well: Based on samples from Europe and North America, they were able to show that connectedness to nature seems to have a positive impact on biospheric value orientation and that biospheric value orientation might mediate the influence of connectedness to nature on behavior. Ojea and Loureiro (2007) found evidence that egoistic as well as altruistic value orientations may increase the financial support of a wildlife protection program. In general, nature conservation measures can be justified by all three value orientations. Thus, altruistic value orientations are linked to the desire to preserve recreational landscapes (landscape conservation) as well as the natural foundations of life for future generations (biodiversity conservation). Biospheric value orientations would lead people to support nature conservation policies independently of their usability for humankind (biodiversity conservation), and the personal desire for recreation might motivate people to support landscape conservation policies–in order to maximize the individual outcome (landscape conservation).

Values (or value orientations) and norms have also been analyzed regarding their significance for sustainable behavior and *climate protection behavior*. A number of studies have already shown that personal norms are important predictors of a variety of environmentally friendly behaviors (Hunecke et al., 2001; Nordlund and Garvill, 2003; Thøgersen, 2006; Bamberg et al., 2007). A personal norm (PN) refers to a person’s conviction that a certain behavior is right or wrong. Thus, the central feature of PN is internalization, and PN is independent of the perceived expectations of others or possible social sanctions (Bamberg et al., 2007). A body of studies have provided empirical evidence that environmental value orientations also contribute to the explanation of pro-environmental behaviors (Nordlund and Garvill, 2003; Steg and de Groot, 2012; Steg et al., 2014). Marshall et al. (2019) investigated not only problem awareness but also the importance of value orientation for self-reported environmental behavior in a specific region–the Australian Great Barrier Reef, which is severely affected by climate change. Based on a sample of N = 1,934 inhabitants of this region, an influence on environmental behavior was demonstrated for altruistic and biospheric values. When talking about pro-environmental behavior (and the support of climate action), PN and environmental value orientations do not seem to be independent of each other. For example, studies by Thøgersen and Grunert-Beckmann (1996) as well as Nordlund and Garvill (2003) showed a mediating effect of PN on the influence of values. This is also consistent with the assumptions of the value belief norm model (VBN) proposed by Stern and Dietz (1994) and Stern (2000), and that is rooted in the assumptions of the norm-activation model (Schwartz, 1977; Schwartz and Howard, 1981) and the New Environmental Paradigm (NEP) of Dunlap and Van Liere (1978).

According to Stern’s conception (Stern, 2000), the support of climate protection measures (here, the implementation of or transition to renewable energy technologies and the shift to renewable resources that serve as carbon sinks) can be understood as a facet of pro-environmental behavior. Given the relevance of biospheric values in influencing pro-environmental behavior (Steg and de Groot, 2012; Steg et al., 2014), we would expect biospheric value orientations to be significant motivators of support for policies for both nature conservation and climate protection. However, not only human values but political orientation, too, seem to have significant impacts on concerns about climate change.

### 1.3. The roles of political orientation and global human identity in the acceptance of climate protection measures

Parallel to studies addressing climate-protection-related values and conservation beliefs with respect to climate protection measures, there is also some evidence that political orientation is relevant to the awareness that the climate crisis is a serious problem and the way in which people evaluate climate protection measures. McCright et al. (2016) reviewed studies that analyzed the relationship between political orientation (left–right ideology) and climate change views in the United States and in European Union countries. Compared with the US, organized denial campaigns seem to be less prominent in the EU. Also for Western European countries, there seems to be stronger support for climate action for citizens on the left than for citizens on the right (except for former communist countries). Carrus et al. (2018) also took a closer look at the role that political orientation plays in relation to environmental attitudes and climate change denial. In line with McCright et al. (2016), they too found evidence that a left-wing or liberal political ideology is more likely to lead to pro-environmental behavior and argued for a more complex analysis of the (psychological) conditions for political ideology and the associated effects.

Boulianne and Belland (2022) analyzed the influence of left–right political orientation in a study with a climate-specific focus (e.g., belief in climate change, support for climate policies, trust in science) and were able to show that a right-wing orientation (mediated by trust in the media and science) had a negative impact on belief in climate change. These results are in line with a similar study by Gregersen et al. (2020).

While there was also the expected positive effect of education on belief in and support for climate policies, this effect seemed to be significantly stronger for a left-wing political orientation, whereas it was in some cases not even significant for a right-wing political orientation (Czarnek et al., 2021). Due to a lack of problem awareness and the associated lower salience of climate policies, a more right-wing political orientation appears to have a negative impact on energy-saving behavior (Gregersen et al., 2021) as well as on the acceptance of climate policy measures, such as additional taxation of fossil fuels (Fairbrother et al., 2019). In this context, counterfactual arguments, fake experts, and misinformation can influence public opinion and might be used strategically by interest groups to bolster their own political agenda (Bush, 2020; Bertolotti et al., 2022; Boecher et al., 2022 Schmid-Petri and Bürger, 2022).

Although we would expect political orientation to show an influence on the acceptance of different climate protection measures, it remains unclear which possible lines of conflict are covered by this right–left dichotomy. Since the 1990s, political orientation has been assumed to run along (a) the socioeconomic conflict between social justice (left) and liberal market freedom (right) and (b) the socio-cultural conflict between liberalism/cosmopolitanism (left) and authoritarianism/conservatism (right; Decker, 2019). With regard to the socio-cultural conflict, we see parallels here with the discussion of the idea of a global identity for the context of the perception and assessment of global climate change (Reese et al., 2015; McFarland et al., 2019; Loy et al., 2022).

Global identity can be understood as a specific type of social identity. The social identity perspective attempts to supplement the previous more individualistic research perspective, that focusses on the impact of internal variables like norms, values, and attitudes to explain people’s pro-environmental behavior. The social identity perspective is focused on the relevance of a kind of self-categorization, that means that people define themselves as being part of a group (social identity) and as individuals (Ferguson et al., 2016; Fritsche et al., 2018; Loy et al., 2022). In the context of research on climate change, a specific type of social identity, namely global identity, has become more relevant and reflects the identification with all humanity (for an overview see Loy et al., 2022). People who were more likely to consider themselves global citizens also seemed to take the impact of climate change more seriously (Running, 2013), and were more angry about climate injustice (Barth et al., 2015). Loy and Reese were also able to show that identification with people around the world was positively related to support for climate action (Loy and Reese, 2019). There are already promising findings concerning the importance of global identity for climate action and pro-environmental behavior, although the exact interplay of internal factors such as norms and values, and human identification remains unclear. The results of a meta-analysis by Agostini and Van Zomeren (2021) support the idea of parallel processes.

Above all, political orientation and self-transcending values (e.g., biospheric values) seem to be among the most consistent and meaningful predictors (Hornsey et al., 2016), although they do not seem to be effective in isolated ways. Using data from 16 Western European countries, Smith and Hempel (2022) analyzed the direct and interactive effects of political orientation and human values on attitudes and behaviors related to climate change. They were able to show that the moderating effects of political orientation were strongest for positive self-transcendence and negative conservation values.

### 1.4. Research questions and research aims

Having addressed the relevance of biospheric values, personal norm, and nature protection beliefs to the acceptance of conservation measures or climate protection measures as well as the roles of political orientation and global human identity, we now return to the central issue of this paper, namely the possible value-based conflicts between nature conservation and climate protection regarding the acceptance of concrete climate protection measures. The study presented here does not aim at testing a full-fledged model to explain the socio-political acceptance of climate mitigation measures. Instead, we are interested in illuminating the specific aspect of possible value-based conflicts between nature conservation motives and climate protection motives.

**Figure 1:**
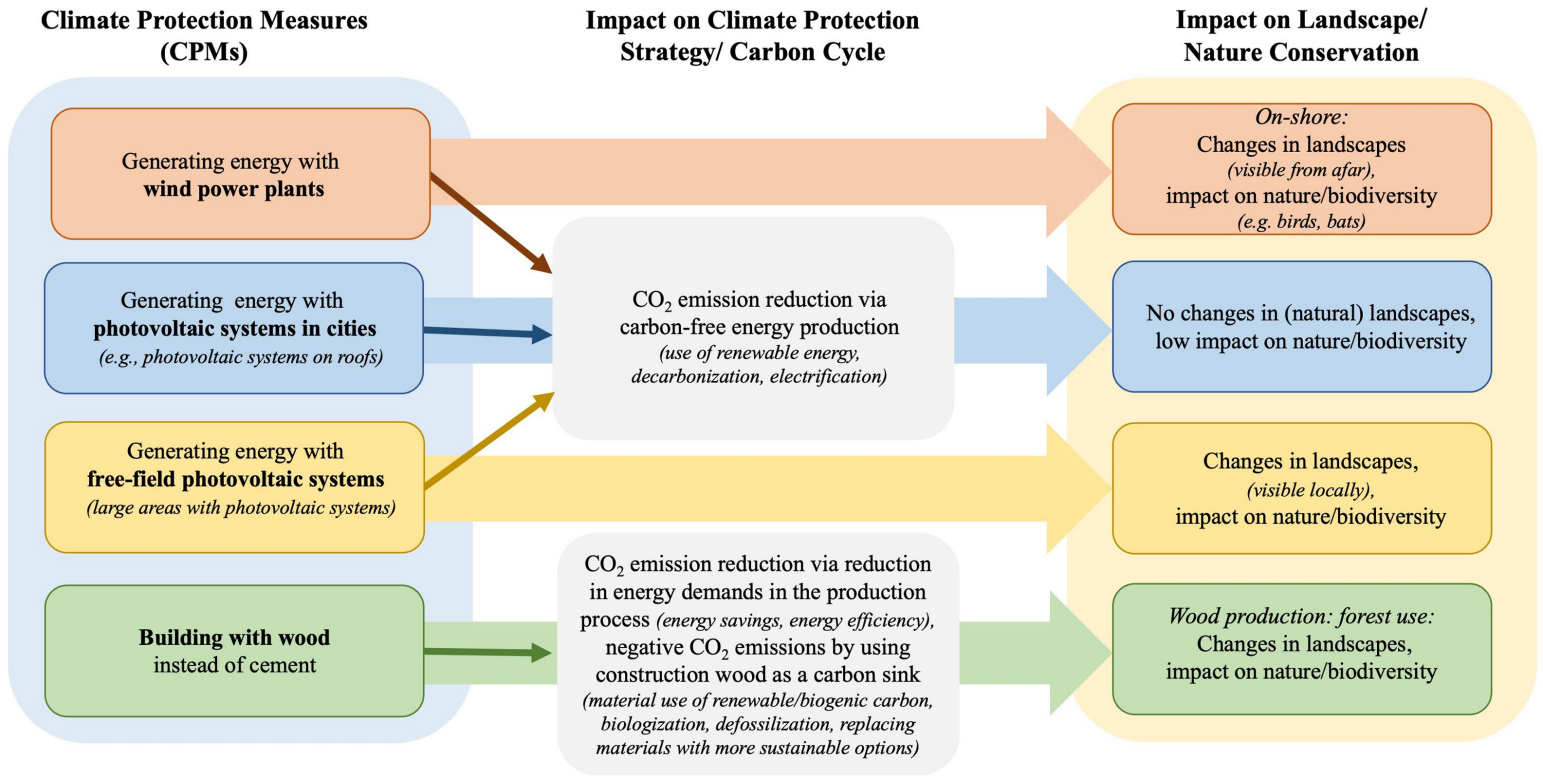
Climate protection measures and their impact on climate and nature.

These potential conflicts might not manifest themselves in the same way for different climate protection measures. In this study, we considered a total of four different measures, some of which have different assumptions (see Figure 1).

We expected that a biospheric value orientation (BV) would have a positive impact on the acceptance of different climate protection measures. We expected this effect for all four of the measures we considered. In this context, we also expected a significant influence of the personal ecological norm (PN) on all measures. In accordance to the assumptions of the VBN we expect mediating effects of PN on the influence of values for all four of the measures. Regarding value orientations and conservation beliefs, the dependencies could be somewhat more complex. Values (defined as guiding principles in life) provide the basis of Nature Conservation Beliefs (NCB). These beliefs are not comparable to the New Environmental Paradigm Scale (NEP) in the context of the VBN. While the NEP reflects a pro-environmental worldview on a more general level, Nature Conservation Beliefs (NCB) are more specific. If nature conservation is primarily based on the preservation of biodiversity as a value in itself, independently of its usability for humankind (biospheric value orientation), this could–although not necessarily–lead to the rejection of measures for the expansion of renewable energies, insofar as these measures are associated with changes in the landscape (energy generation from wind turbines, energy generation from free-field photovoltaic systems) or the associated impairment of habitats (forest management measures for carbon benefits: building with wood instead of cement). In this context, it should be mentioned that in relation to building with wood, not everyone will see the connection between this measure and forest management measures and the interference with existing habitats. If nature conservation is justified by the recreational value of nature, this belief might also conflict with landscape alteration measures (generating energy with wind turbines, generating energy with free-field photovoltaic systems). Even if building with wood also has landscape-altering consequences due to the necessary forestry management measures, there is not necessarily a conflict here because this effect might not be represented at all.

As pointed out, there is some evidence that political orientation and humanity orientation can have an influence on the acceptance of climate protection measures. In a similar way that values form the basis for conservation beliefs and for norms, we also assume that political orientations and human identity function in the sense of a fundamental, context-independent guideline and influence conservation beliefs and norms. The present study is aimed at illuminating in more detail the possible interrelationships between political orientation, global human identity, and both nature-conservation-related and climate-protection-related beliefs and value orientations in order to identify and better understand possible underlying value-based conflicts.

## 2. Materials and methods

### 2.1. Participants and procedure

The data reported here are part of a larger survey involving an interdisciplinary group of researchers. The overall survey examined climate change mitigation strategies, climate change mitigation behaviors, and political support for climate change mitigation technologies. In the following, only the survey measures that have been implemented to analyze possible conflicts between nature conservation motives and climate protection motives in the scope of the acceptance of climate protection measures are presented in more detail. The data were collected in an online survey from April 22nd to May 19th, 2022, Germany-wide. The company Bilendi was commissioned to recruit a stratified sample.^2^ *N =* 4,600 people visited the link and 1,584 finished the online questionnaire. The sample was stratified to be representative of the German population regarding age (for the 16 to 74 olds), gender, education, and place of residence, reflecting the distributions of those characteristics in the general German population. The criterion “place of residence” was based on the distribution statistics of the German population according to municipality size classes (Statistisches Bundesamt, 2023) to realize a representative distribution of the sample with regard to urban–rural areas. After unreliable cases were excluded on the basis of participants’ answering time, missing values, and unrealistic answers to open questions, a total of *N* = 1,427 participants formed the final sample. Participants’ age ranged from 16 to 74 (*M* = 47.18, *SD* = 15.94). 50.2% were female, 49.7% were male, and 0.1% identified as diverse. Regarding participants’ highest level of education, 1.9% did not have a school degree (yet), 6.3% reported a secondary school diploma but had not completed an apprenticeship, 31.4% reported a secondary education, 24.7% reported a secondary school diploma and had completed an apprenticeship, 17% had a higher-education entrance qualification, and 18.6% had completed higher education.

**Figure 2:**
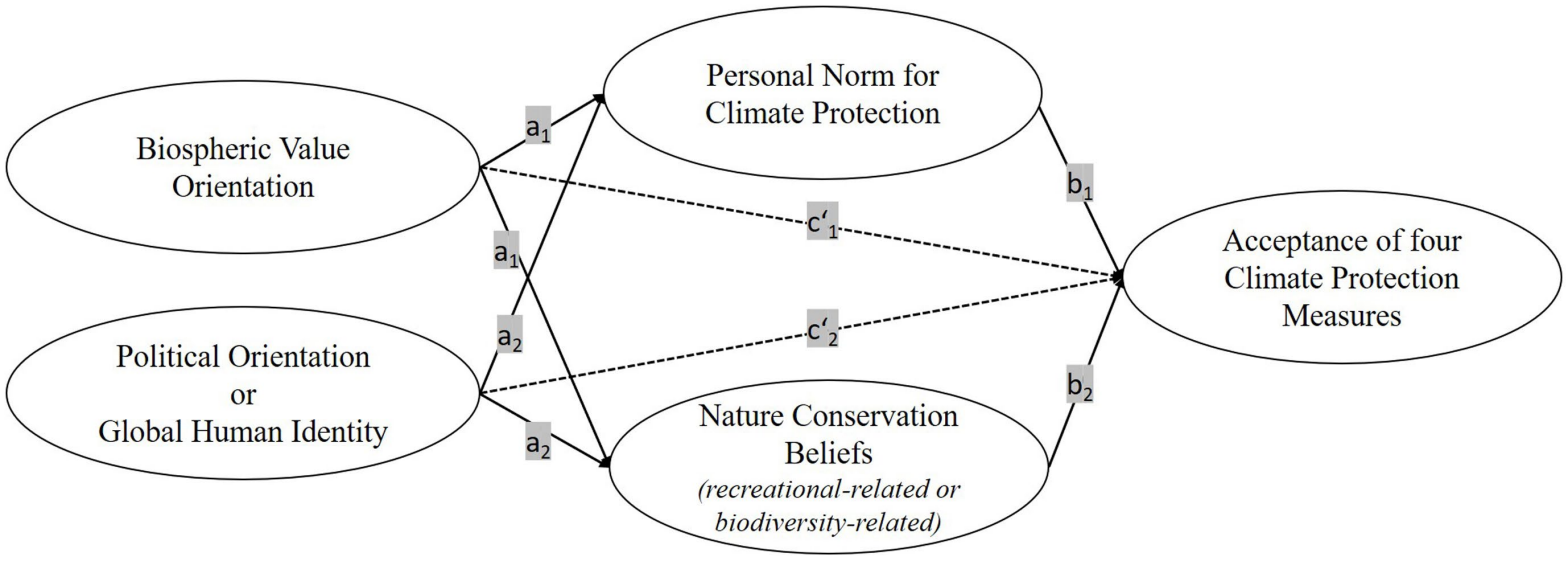
Path Diagram for the Mediation Model. a = direct effect of predictor on mediator, b = direct effect of mediator on criterion ( a×b = indirect effect), $c^{\prime }$ = direct effect of predictor on criterion. The four climate protection measures were: generating energy with wind turbines, generating energy with photovoltaic systems in cities, generating energy with free-field photovoltaic systems, building with wood instead of cement.

Because some psychological variables showed high percentages of missing data, we conducted a missing value analysis. As Little’s MCAR-Test (Little, 1988); *χ*^2^ (141161) = 143518.41, *p* < 0.001 suggested that the missing data were on a continuum between missing at random and not missing at random and as the missingness of the data was moderately to highly correlated with other variables, the estimation maximization method with NORM Version 2.03 was chosen as an appropriate method for imputation (Schafer, 1999). The items measuring the acceptance of climate protection measures were excluded from the imputation because only participants who knew those measures answered the respective questions. Figure 2 provides an overview of the mediation hypotheses.

### 2.2. Measures

#### 2.2.1. Personal norm for climate protection

We measured participants’ personal norm (PN) for climate protection with three items taken from Matthies and Merten (2022) with reference to Schwartz and Howard (1981). The three items (“No matter what other people think, it is important to me to get involved in climate protection”; “Because of my values, it is important to me to support climate protection measures”; “I feel obligated to save CO2 in my everyday life”) were answered on a 5-point Likert scale (1 = completely disagree; 5 = completely agree). We choose to measure a rather broad PN concept focusing on individual climate protective behavior to predict a range of differently characterized policy measures. The items had high reliability (ω = 0.93) and large standardized factor loadings (0.87 ≤ λ ≤ 0.93; in a unidimensional CFA with robust Maximum Likelihood (MLR) estimation).

#### 2.2.2. Biospheric value orientation

Participants’ biospheric value orientation was measured with three items from the Brief Inventory of Values by Stern et al. (1998) (“Protecting the environment, preserving nature”; “Respecting the earth, harmony with other species”; “Unity with nature, fitting into nature”). Each item was rated as a “guiding principal in my life” on a 9-point Likert scale (−1 = opposed to my values; 0 = not important, 1 = unlabeled, 2 = unlabeled, 3 = important, 4 = unlabeled, 5 = unlabeled, 6 = very important, 7 = extremely important), that has already been used by Schwartz (1992).^3^ These items showed a sufficiently high reliability (*ω* = 0.87), and the unidimensional CFA^4^ yielded appropriate standardized factor loadings (0.78 ≤ *λ* ≤ 0.88; MLR estimation).

#### 2.2.3. Nature conservation beliefs

We measured two different nature conservation beliefs, each referring to a different value orientation. The two beliefs were NCB_recr: “The key task of nature conservation lies in the preservation of human recreational spaces” (based on altruistic values: recreational value of nature) and NCB_biodiv: “The key task of nature conservation is to safeguard the biodiversity of animals and plants” (based on biospheric value orientation/conservation as a value itself). These beliefs were measured with single items^5^ that were answered on a 5-point Likert scale (1 = completely disagree; 5 = completely agree).

#### 2.2.4. Global human identity

To measure participants’ global human identity, we developed a short version of the nine-item IWAH scale (identification with all humanity), originally developed by McFarland et al. (2012) and translated into a German version by Loy et al. (2022) and Reese et al. (2015). The short version was slightly modified and consisted of four items (“I feel connected to people all over the world”; “I think of people all over the world as ‘we’”; “I feel in solidarity with people all over the world”; “I want to be a responsible member of the global community”), which were scored on a 7-point Likert scale (1 = does not apply at all; 7 = fully applies). The scale was highly reliable (*ω* = 0.93), and the unidimensional CFA yielded appropriate standardized factor loadings (0.77 ≤ *λ* ≤ 0.91; MLR estimation) as well as fit indices [robust CFI = 0.999, robust RMSEA = 0.045, SRMR = 0.006; (Hu and Bentler, 1999)].

#### 2.2.5. Political orientation

Participants’ political orientation was measured with a left–right self-placement scale consisting of only a single item (“Many people use the terms ‘left’ and ‘right’ when referring to different political attitudes. When you think about your political views, where would you rank those views on this scale?”) on which they could rank themselves on a scale ranging from 1 (left) to 10 (right). This measure allowed respondents to make a classification intuitively and without prior expertise. Even if using a simplified dualism left open what the respondents associated with the terms left and right, studies have shown that most respondents are able to locate both themselves and political parties on the left–right scale (Mair, 2007). This scale is well established for the use on German samples: It is regularly used in election research for Germany, and in political surveys, such as the German General Social Survey (ALLBUS) (Breyer, 2015).

#### 2.2.6. Acceptance of climate protection measures

To identify potential conflicts between nature protection and climate protection, participants were asked for their acceptance of four different climate action measures. These were selected because of their potential to illuminate the conflict between nature protection and climate protection: (1) Generating energy with wind turbines, (2) Generating energy with photovoltaic systems in cities (e.g., photovoltaic systems on roofs), (3) Generating energy with free-field photovoltaic systems (large areas with photovoltaic systems), and (4) Building with wood instead of cement. These four items measured participants’ attitude toward each particular strategy on a 5-point Likert scale (1 = completely disagree; 5 = completely agree). Item 2 plays a special role here, as photovoltaic installations in cities are not associated with landscape-altering effects (recreational dimension) and also do not have a clear direct impact on biodiversity (biodiversity dimension). Whether Item 4 is connected to having an impact on biodiversity will largely depend on whether people are aware of the associated forestry measures.

## 3. Results

We conducted the main analysis with R version 4.0.3 (R Core Team, 2020) and the following R-packages: dplyr (Wickham et al., 2021), EFAtools (Steiner and Grieder, 2020), lavaan (Rosseel, 2012), MVN (Korkmaz et al., 2014), nortest (Gross and Ligges, 2015), pastecs (Grosjean and Ibanez, 2018), psych (Revelle, 2020), semhelpinghands (Cheung, 2023), and utils (R Core Team, 2020). All R scripts are available from XY upon request. We used IBM SPSS Statistics 27 (IBM Corporation, 2020) to prepare the data.

First, we computed basic descriptive and bivariate statistics. Next, we tested structural equation models (SEM) to establish the predictor structure of the CPM which were used as criteria. Modelling the predictor structure, we chose a path configuration that is both in line with theoretical assumptions and the intention to preliminarily investigate an intrapersonal conflict (Gunzler et al., 2013; Danner et al., 2015). Based on previous research on the relation between personal norms and values (e.g., Joireman and Liu, 2014; van der Werff and Steg, 2016; Wynveen and Sutton, 2017), we hypothesized that broad, fundamental, and comparatively stable concepts such as biospheric values and political orientations inform the development of more specific psychological constructs such as personal norms and beliefs. Furthermore, we specifically operationalized the NCB to be justified by different values (biospheric and anthropocentric values). Hence, PN and NCB were assumed to be mediators of BV and PO or GHI. To obtain first evidence of a potential conflict between climate protection orientation and nature protection orientation, the CPM were directly regressed on PN and both NCB (potentially yielding contrary regression coefficients) while controlling for the influence of BV and PO or GHI. Figure 2 provides an overview of the corresponding path diagram. We estimated a total of eight SEMs in which PN and NCBs both mediated the effects of BV and either PO or GHI (only one of the two constructs was used as the predictor per SEM) on the four different CPM items.

A simple mediation model with a predictor *X* whose effect on a criterion Y is mediated by a third variable (the mediator *M*) can be described by three regression models. First, the total effect of the predictor *X* is estimated by regressing the criterion *Y* on the predictor *X* (Equation 1). Then, the mediator *M* is regressed on the predictor *X* (Equation 2), while the criterion *Y* is simultaneously regressed on the predictor *X* and the mediator *M* (Equation 3). In the equations i represents the intercept of the model and e the error.

(1) $$Y_{i}=i_{Y.X}+cX_{i}+e_{Y.X_{i}}$$

(2) $$M_{i}=i_{M.X}+aX_{i}+e_{M.X_{i}}$$

(3) $$Y_{i}=i_{Y.MX}+bM_{i}+c^{\prime }X_{i}+e_{Y.MX_{i}}$$

We refrained from interpreting the total effect c from Equation (1) as it can be misleading and as its significance is not indicative of the existence a mediation effect (Zhao et al., 2010; Rucker et al., 2011; Igartua and Hayes, 2021). The effect of predictor *X* on criterion *Y* consists of two additive paths ( $c=c^{\prime }+ab$ ). The indirect effect of *X* on *Y* through mediator M equals the product of a and b from Equations (2, 3). The second path is the direct effect $c^{\prime }$ of *X* on *Y* controlled for *M*. Thus, $c^{\prime }$ captures the effects independent of the mediation. When assessing whether a mediation is significant, the pattern of c and $c^{\prime }$ is irrelevant (Igartua and Hayes, 2021). We only focused on the indirect effect ab (how *X* affects *Y* by affecting *M*) and assumed a mediation when this product was significant and when the confidence interval did not include zero. The previous distinction into full and partial mediation advocated by Baron and Kenny (1986) will not be considered as it relies on the interpretation of the pattern of c and $c^{\prime }$ . Nevertheless, a significant indirect effect $c^{\prime }$ can indicate the existence of additional mediators (Zhao et al., 2010).

The parameters of these SEMs were estimated with a Maximum Likelihood (ML) algorithm combined with the bootstrapping of 1,000 samples (Igartua and Hayes, 2021).To estimate the confidence intervals (CI) of the parameters and account for the non-normal distribution of the indirect effects in a mediation analysis, we computed the 95% bootstrap percentile CI. For the single-item measures, a latent variable was defined with the item loading on it having an unstandardized error variance of 0.10 (reflecting a “scale” reliability of 0.90).

In the description of the results, we refrained from using causal language, as our cross-sectional design does not allow the interpretation of the found relations between the variables as causal mechanisms.

### 3.1. Descriptive and bivariate statistics

Supplementary Table A1 provides an overview of the item-level descriptive statistics. Both tests of univariate normality – the Shapiro–Wilk test and the Shapiro-Francia test – indicated a deviation from normality for all items (Shapiro and Wilk, 1965; Royston, 1983). Furthermore, the item groups that formed the scales for measuring PN, BV, and GHI were not multivariate normally distributed (Mardia, 1970; Henze and Zirkler, 1990); see Suppelementary Tables A3–A6 for the respective test statistics.

The means, medians, standard deviations, skewness, and kurtosis of the manifest factor scores (based on the mean of the items) and single-item measures can be found in Supplementary Table A2. Again, the Shapiro–Wilk and the Shapiro-Francia tests of univariate normality indicated that none of the manifest factor scores or single-item measures were normally distributed (see Suppelementary Tables A3–A6). The means of all constructs were significantly^6^ greater than the scale midpoints except for the mean of recreational NCB (*M* = 2.98). In particular, the four CBM measures were highly skewed toward high acceptance (*M_Wind Turbines_* = 4.19; *M_Photovoltaic (city)_* = 4.55; *M_Photovoltaic (free-field)_* = 4.18; *M_Building with Wood_* = 3.91), thus indicating that the participants who knew about these measures also accepted them to a large degree. Furthermore, the means of both GHI (*M* = 4.61) and PO (*M* = 5.93) were slightly skewed toward higher values, thus reflecting that our sample was more politically oriented toward the left and tended to identify with all of humankind.

The Pearson correlation coefficients can be found in Table 1. Applying the common effect size cut-offs for Pearson correlation coefficients, the four CPM items were correlated to a small or medium degree (0.13 ≤ *r* ≤ 0.38) with all constructs but recreational NCB (−0.07 ≤ *r* ≤ −0.01). Furthermore, the correlation between the two NCB measures was rather small (*r* = 0.06), thus supporting the assumption that these beliefs refer to different value orientations (biospheric versus altruistic value orientation). The correlations between biodiversity-related NCB and PN (*r* = 0.34), BV (*r* = 0.49), PO (*r* = 0.13), as well as GHI (*r* = 0.28) were significantly^7^ larger than the correlations between recreation-related NCB and these constructs (*r* = 0.09, *r* = 0.15, *r* = −0.07, *r* = 0.16). The acceptance of generating energy with photovoltaic systems in cities and of building with wood instead of cement were also significantly more strongly associated with GHI (*r_Photovoltaic (city)_* = 0.25, *r_Building with Wood_* = 0.26) than with PO (*r_Photovoltaic (city)_* = 0.14, *r_Building with Wood_* = 0.13), whereas the difference between the correlations was not significant for the acceptance of generating energy with wind turbines or generating energy with free-field photovoltaic systems.

**Table 1:** Pearson correlation coefficients based on manifest scale scores.

	BV	NCB		GHI	PO		CPM		
		Recreational	Biodiversity			WT	PV (city)	PV (field)	BW
PN	0.56^***^ (1427)	0.09^***^ (1427)	0.34^***^ (1427)	0.60^***^ (1427)	0.30^***^ (1297)	0.38^***^ (1280)	0.32^***^ (1219)	0.29^***^ (1042)	0.27^***^ (1093)
BV		0.15^***^ (1427)	0.49^***^ (1427)	0.48^***^ (1427)	0.16^***^ (1297)	0.22^***^ (1280)	0.29^***^ (1219)	0.18^***^ (1042)	0.26^***^ (1093)
*NCBs*									
NCB (recreational)			0.06^*^ (1427)	0.16^***^ (1427)	−0.07^*^ (1297)	−0.05 (1280)	−0.01 (1219)	−0.02 (1042)	−07^*^ (1093)
NCB (biodiversity)				0.28^***^ (1427)	0.13^***^ (1297)	0.17^***^ (1280)	0.29^***^ (1219)	0.17^***^ (1042)	0.18^***^ (1093)
GHI					0.28^***^ (1297)	0.26^***^ (1280)	0.25^***^ (1219)	0.20^***^ (1042)	0.26^***^ (1093)
PO						0.22^***^ (1182)	0.14^***^ (1134)	0.16^***^ (981)	0.13^***^ (1014)
*CPMs*									
Wind Turbines							0.46^***^ (1177)	0.52^***^ (995)	0.20^***^ (1033)
Photovoltaic (city)								0.49^***^ (1000)	0.24^***^ (999)
Photovoltaic (free-field)									0.23^***^ (899)

PN, Personal Norm for Climate Protection; BV, Biospheric Value Orientation; NCBs, Nature conservation beliefs; GHI, Global Human Identity; PO, Political Orientation; CPMs, Acceptance of Climate Protection Measures, WT, Generating energy with wind turbines, PV (city), Generating energy with photovoltaic systems in cities, PV (field), Generating energy with free-field photovoltaic systems; BW, Building with wood instead of cement. Pairwise deletion was applied. *n* in parentheses. ^*^p < 0.05, ^**^p < 0.01, ^***^p < 0.001.

### 3.2. Mediation analyses

The eight SEMs that we computed to test our mediation hypotheses all showed good to excellent model fit (CFI ≥ 0.967, RMSEA ≤0.072, SRMR ≤0.044; Hu and Bentler, 1999) despite the significant *χ*^2^ test statistics oversensitive in large samples; (Bentler and Bonett, 1980). Table 2 provides an overview of the fit indices.

**Table 2:** Fit Indices for the eight structural equation models for testing the mediation hypotheses.

Model	CFI	RMSEA	SRMR	BIC	*χ^2^* (df)
*Criterion: CPM Wind Turbines*					
With Political Orientation (*n* = 1,182)	0.981	0.059	0.028	34,306	137.1^***^ (27)
With Global Human Identity (*n* = 1,280)	0.969	0.069	0.044	47,103	398.5^***^ (56)
*Criterion: CPM Photovoltaic (city)*					
With Political Orientation (*n* = 1,134)	0.975	0.068	0.031	32,067	168.3^***^ (27)
With Global Human Identity (*n* = 1,219)	0.967	0.072	0.043	43,831	408.2^***^ (56)
*Criterion: CPM Photovoltaic (free-field)*					
With Political Orientation (*n* = 981)	0.982	0.056	0.027	28,504	111.0^***^ (27)
With Global Human Identity (*n* = 1,042)	0.967	0.071	0.042	38,314	348.4^***^ (56)
*Criterion: CPM Building with wood*					
With Political Orientation (*n* = 1,014)	0.985	0.051	0.024	29,272	99.2^***^ (27)
With Global Human Identity (*n* = 1,093)	0.972	0.065	0.041	39,898	317.5^***^ (56)

*χ*^2^ = *χ*^2^ test statistic, df, Degrees of Freedom; CFI, Comparative Fit Index; RMSEA, Root Mean Square Error of Approximation; SRMR, Standardized Root Mean Square Residual; BIC, Bayesian Information Criterion; CPM, Climate Protection Measures. ^***^ *p* < 0.001. Maximum Likelihood was applied.

**Figure 3:**
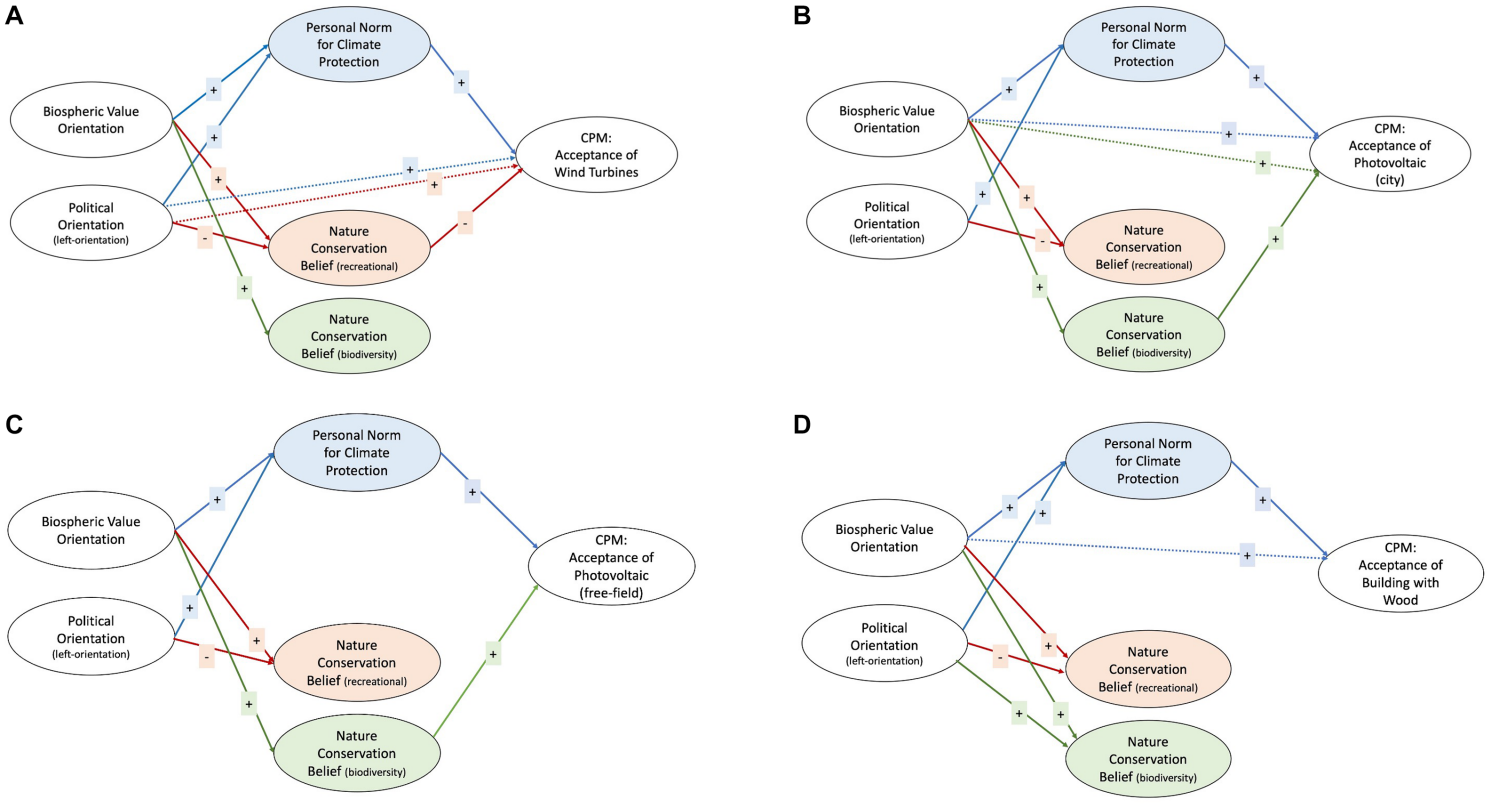
Overview of the Results for the Mediation Hypotheses with Political Orientation. Note. **(A)** Wind turbines, **(B)** Photovoltaic (city), **(C)** Photovoltaic (free-field), and **(D)** Building with Wood.

As we achieved a model fit that was sufficient for the further interpretation of the models, we now turn to the different model paths. Tables 3 4 present the path coefficients and *R*^2^ values for all SEMs, while Figure 3 and Table 5 contain the overall interpretations of the models.

**Table 3:** Structural equation models for testing the mediation hypotheses with biospheric value orientation (BV) and political orientation (PO) as predictors.

Model	*β* (95%-CI)					Completely Standardized Indirect Effect (95%-CI) (BV→)	Completely Standardized Indirect Effect (95%-CI) (PO→)	*R* ^2^
	a_1_ (BV→)	a_2_ (PO→)	b	c’	c			
CPM Wind Turbines (*n* = 1,182)								0.19
BV				0.03 (−0.09; 0.13)	0.24^***^ (0.16; 0.31)			
PO				0.10^**^ (0.03; 0.15)	0.18^***^ (0.12; 0.24)			
PN	0.59^***^ (0.54; 0.63)	0.21^***^ (0.17; 0.26)	0.36^***^ (0.27; 0.45)			0.21^***^ (0.16; 0.26)	0.08^***^ (0.05;0.11)	
NCB (recreational)	0.15^***^ (0.08; 0.22)	−0.10^**^ (−0.16; −0.04)	−0.10^**^ (−0.16; −0.04)			−0.02^**^ (−0.03; −0.01)	0.01^*^ (0.003;0.02)	
NCB (biodiversity)	0.53^***^ (0.47; 0.58)	0.03 (−0.03; 0.09)	0.04 (−0.05; 0.13)			0.02 (−0.03; 0.07)	0.001 (−0.003; 0.01)	
								
CPM Photovoltaic (city) (*n* = 1,134)								0.21
BV				0.13^*^ (0.004; 0.27)	0.36^***^ (0.28; 0.43)			
PO				0.04 (−0.03; 0.10)	0.09^**^ (0.03; 0.16)			
PN	0.59^***^ (0.54; 0.64)	0.21^***^ (0.17; 0.26)	0.20^***^ (0.09; 0.30)			0.12^***^ (0.05; 0.18)	0.04^**^ (0.02; 0.07)	
NCB (recreational)	0.16^***^ (0.09; −22)	−0.09^**^ (−0.15; −0.03)	−0.06 (−0.12;-0.004)			−0.01 (−0.02; −0.001)	0.01 (0.00; 0.01)	
NCB (biodiversity)	0.54^***^ (0.48; 0.59)	0.04 (−0.02; 0.10)	0.21^***^ (0.12; 0.30)			0.11^***^ (0.07; 0.16)	0.01 (−0.004; 0.02)	
								
CPM Photovoltaic (free-field) (*n* = 981)								0.11
BV				−0.02 (−0.16; 0.10)	0.18^***^ (0.10; 0.25)			
PO				0.07 (−0.003; 0.13)	0.14^***^ (0.07; 0.20)			
PN	0.59^***^ (0.54; 0.64)	0.23^***^ (0.17; 0.28)	0.27^***^ (0.16; 0.38)			0.16^***^ (0.09; 0.23)	0.06^***^ (0.03; 0.09)	
NCB (recreational)	0.15^***^ (0.08; 0.23)	−0.10^**^ (−0.17; −0.03)	−0.06 (−0.12; 0.01)			−0.01 (−0.02; 0.002)	0.01 (−0.001; 0.02)	
NCB (biodiversity)	0.54^***^ (0.47; 0.60)	0.02 (−0.04; 0.09)	0.11^*^ (0.01; 0.20)			0.06^*^ (0.01; 0.11)	0.002 (−0.01; 0.01)	
								
CPM Building with wood (*n* = 1,014)								0.11
BV				0.14^*^ (0.03; 0.26)	0.27^***^ (0.20; 0.35)			
PO				0.05 (−0.01; 0.11)	0.09^**^ (0.02; 0.15)			
PN	0.60^***^ (0.54; 0.65)	0.20^***^ (0.15; 0.25)	0.17^**^ (0.06; 0.27)			0.10^**^ (0.04; 0.16)	0.03^**^ (0.01; 0.06)	
NCB (recreational)	0.14^***^ (0.08; 0.22)	−0.10^**^ (−0.16; −0.04)	0.04 (−0.03; 0.11)			0.01 (−0.004; 0.02)	−0.004 (−0.01; 0.003)	
NCB (biodiversity)	0.51^***^ (0.45; 0.57)	0.06^*^ (0.001; 0.12)	0.05 (−0.04; 0.14)			0.03 (−0.02; 0.07)	0.003 (−0.003; 0.01)	

β, standardized regression coefficient; CI, confidence interval, *R*^2^, amount of variance explained in the criterion by all predictors and mediators, a, effect of predictor on mediator, b, effect of mediator on criterion, $c^{\prime },$ direct effect of predictor on criterion, c , total effect of predictor on criterion, PN, Personal Norm for Climate Protection, BV, Biospheric Value Orientation, NCB, Nature Conservation Belief, PO, Political Orientation, CPM, Acceptance of Climate Protection Measure. Maximum Likelihood estimation and bootstrapping were applied. ^*^p < 0.05, ^**^p < 0.01, ^***^p < 0.001.

**Table 4:** Structural equation models for testing the mediation hypotheses with biospheric value orientation (BV) and global human identity (GHI) as predictors.

Model	*β* (95%-CI)					Completely Standardized Indirect Effect (95%-CI) (BV→)	Completely Standardized Indirect Effect (95%-CI) (GHI→)	*R^2^*
	a_1_ (BV→)	a_2_ (GHI→)	b	c’	c			
CPM Wind Turbines (*n* = 1,280)								0.18
BV				0.004 (−0.11; 0.11)	0.18^***^ (0.09; 0.27)			
GHI				0.05 (−0.03; 0.13)	0.19^***^ (0.10; 0.27)			
PN	0.41^***^ (0.35; 0.47)	0.43^***^ (0.36; 0.47)	0.37^***^ (0.28; 0.46)			0.15^***^ (0.11; 0.20)	0.16^***^ (0.11; 0.21)	
NCB (recreational)	0.07 (−0.001; 0.15)	0.13^**^ (0.06; 0.20)	−0.11^***^ (−0.16; −0.05)			−0.01 (−0.02; 0.00)	−0.01^*^ (−0.03; −0.004)	
NCB (biodiversity)	0.56^***^ (0.48; 0.63)	−0.01 (−0.09; 0.06)	0.06 (−0.02; 0.14)			0.03 (−0.01; 0.08)	−0.001 (−0.01; 0.004)	
CPM Photovoltaic (city) (*n* = 1,219)								0.20
BV				0.12 (−0.004; 0.26)	0.32^***^ (0.23; 0.41)			
GHI				0.02 (−0.06; 0.11)	0.10^*^ (0.02; 0.19)			
PN	0.41^***^ (0.35; 0.48)	0.43^***^ (0.36; 0.49)	0.21^***^ (0.09; 0.32)			0.09^***^ (0.04; 0.13)	0.09^**^ (0.04; 0.14)	
NCB (recreational)	0.08^*^ (0.003; 0.15)	0.13^**^ (0.06; 0.21)	−0.06^*^ (−0.11; −0.01)			−0.01 (−0.01; 0.00)	−0.01 (−0.02; −0.001)	
NCB (biodiversity)	0.57^***^ (0.49; 0.64)	−0.02 (−0.09; 0.06)	0.20^***^ (0.12; 0.28)			0.11^***^ (0.07; 0.17)	−0.003 (−0.02; 0.01)	
CPM Photovolt. (free-field) (*n* = 1,042)								0.10
BV				−0.02 (−0.14; 0.11)	0.15^**^ (0.05; 0.25)			
GHI				0.02 (−0.08; 0.12)	0.13^**^ (0.03; 0.22)			
PN	0.42^***^ (0.35; 0.48)	0.42^***^ (0.35; 0.49)	0.28^***^ (0.17; 0.39)			0.12^***^ (0.07; 0.17)	0.12^***^ (0.07; 0.17)	
NCB (recreational)	0.06 (−0.02; 0.14)	0.15^***^ (0.07; 0.22)	−0.05 (−0.12; 0.02)			−0.003 (−0.01; 0.002)	−0.01 (−0.02; 0.002)	
NCB (biodiversity)	0.56^***^ (0.47; 0.63)	−0.01 (−0.09; 0.06)	0.10^*^ (0.01; 0.19)			0.06^*^ (0.003; 0.11)	−0.001 (−0.01; 0.01)	
CPM Building with wood (*n* = 1,093)								0.12
BV				0.11 (−0.01; 0.22)	0.20^***^ (0.11; 0.29)			
GHI				0.11^*^ (0.01; 0.20)	0.17^***^ (0.08; 0.25)			
PN	0.43^***^ (0.36; 0.49)	0.40^***^ (0.33; 0.47)	0.13^*^ (0.03; 0.24)			0.06^*^ (0.01; 0.10)	0.05^*^ (0.01; 0.10)	
NCB (recreational)	0.05 (−0.02; 0.13)	0.13^**^ (0.06; 0.21)	0.04 (−0.03; 0.10)			0.002 (−0.002; 0.01)	0.01 (−0.004; 0.02)	
NCB (biodiversity)	0.52^***^ (0.44; 0.59)	0.02 (−0.05; 0.10)	0.07 (−0.02; 0.15)			0.03 (−0.01; 0.08)	0.001 (−0.01; 0.01)	

β, standardized regression coefficient; CI, confidence interval; *R*^2^, amount of variance explained in the criterion by all predictors and mediators, a , effect of predictor on mediator; b , effect of mediator on criterion; $c^{\prime }$ , direct effect of predictor on criterion; c , total effect of predictor on criterion; PN, Personal Norm for Climate Protection; BV, Biospheric Value Orientation; NCB, Nature Conservation Belief; GHI, Global Human Identity; CPM, Acceptance of Climate Protection Measure. Maximum Likelihood estimation and bootstrapping were applied. ^*^p < 0.05, ^**^p < 0.01, ^***^p < 0.001.

**Table 5:** Overview of the results for the mediation hypotheses with biospheric value orientation (BV) and global human identity (GHI).

Criterion	Mediator	Predictor	
		BV	GHI
CPM wind turbines	PN	mediation	mediation
	NCB recreational	no mediation	mediation
	NCB biodiversity	no mediation	no mediation
			
CPM Photovoltaic (city)	PN	mediation	mediation
	NCB recreational	no mediation	no mediation
	NCB biodiversity	mediation	no mediation
			
CPM Photovoltaic (free-field)	PN	mediation	mediation
	NCB recreational	no mediation	no mediation
	NCB biodiversity	mediation	no mediation
			
CPM building with wood	PN	mediation	mediation
	NCB recreational	no mediation	no mediation
	NCB biodiversity	no mediation	no mediation

PN, Personal Norm for Climate Protection, BV, Biospheric Value Orientation, NCB, Nature conservation beliefs, GHI, Global Human Identity, CPM, Acceptance of Climate Protection Measure.

### 3.3. Acceptance of wind turbines

Turning first to the model with BV and PO as the predictors and wind turbines as the criterion, PN mediated the relationship between BV and the acceptance of wind turbines. A higher level of BV was positively associated with PN, whereas PN had a positive relationship with acceptance. This relationship was the same for PO (being more politically oriented to the left was positively associated with PN) with the difference that PO was still a significant predictor of acceptance when the effect of PN was controlled for. Furthermore, we found that recreational NCB mediated both BV and PO’s relationships with the acceptance of wind turbines. A higher BV was associated with a higher recreational NCB, which was negatively associated with the acceptance of wind turbines. Also, being more politically oriented toward the right was positively associated with recreational NCB, whereas recreational NCB had a negative relationship with the acceptance of wind turbines. We did not find any mediating effect of biodiversity-related NCB in this model.

The model with BV and GHI as predictors yielded a similar pattern with PN mediating both the relationship between BV and the acceptance of wind turbines and the relationship between GHI and acceptance (same associations as in the model with PO). We found no mediating effect of recreational NCB on the association between BV and the acceptance of wind turbines, but recreational NCB mediated the relationship between GHI and acceptance. For participants with a higher GHI, it was more important to protect nature for recreational reasons, and this higher level of recreational NCB had a negative association with the acceptance of wind turbines. Again, we found no mediating effect of biodiversity-related NCB.

The *R*^2^ (in our case, the amount of variance explained in the CPM item) was 0.19 in the model with PO and 0.18 in the model with GHI.

### 3.4. Acceptance of photovoltaic systems in cities

In the model with BV and PO as the predictors and the acceptance of photovoltaic systems (PVS) in cities as the criterion, we found that PN mediated the relationship between BV and the acceptance of PVS in cities, while the direct relation of BV and PVS in cities was still significant and positive. BV was positively associated with PN, which in turn had a positive relationship with the acceptance of PVS in cities. PN also mediated the relationship between PO and acceptance. By contrast, we found no mediating effect of recreational NCB in this model. Biodiversity-related NCB mediated the relationship between BV and the acceptance of PVS in cities but not the relationship between PO and acceptance. A higher BV was positively associated with the belief that nature conservation should secure biodiversity, and higher biodiversity-related NCB was associated with a higher acceptance of PVS in cities.

We found the same mediating effects in the model with BV and GHI as the predictors (without a significant direct relation of BV). To elaborate on the mediation involving GHI, GHI was positively associated with PN, which in turn had a positive effect on the acceptance of PVS in cities.

The *R*^2^ was 0.21 in the model with PO and 0.20 in the model with GHI.

### 3.5. Acceptance of free-field photovoltaic systems

In the model with BV and PO as the predictors, we found that PN had a mediating effect on BV and PO’s relationships with the acceptance of free-field PVS. A higher level of BV and being more politically oriented to the left were positively associated with PN, which in turn had a positive relationship with acceptance. We found no mediating effect of recreational NCB, but biodiversity-related NCB mediated the relationships between BV and the acceptance of PVS on free fields. BV had a positive relationship with biodiversity-related NCB, which in turn had a positive association with the acceptance of free-field PVS.

Again, the model with BV and GHI as the predictors yielded the same mediation effects. A higher level of GHI was positively related to PN, which had a positive association with the acceptance of free-field PVS.

The *R*^2^ was 0.11 in the model with PO and 0.10 in the model with GHI.

### 3.6. Acceptance of building with wood instead of cement

We found that PN mediated BV and PO’s relationships with the acceptance of building with wood instead of cement. Whereas having a higher BV and being more politically oriented to the left were positively related to PN, a higher PN was positively associated with the acceptance of building with wood instead of cement. We found no other mediating effects in the model.

The same mediation pattern applied to the model with BV and GHI as the predictors. A higher GHI had a positive relationship with PN, and a higher PN was positively associated with the acceptance of building with wood instead of cement.

The *R*^2^ was 0.11 in the model with PO and 0.12 in the model with GHI.

## 4. Discussion

In the study at hand, we were interested in investigating possible value-based conflicts between motives for nature conservation and motives for climate protection regarding the acceptance of climate protection measures. It was expected that these potential conflicts might not manifest themselves in the same way for different climate protection measures. Furthermore, the study was aimed at illuminating possible interrelationships between political orientation (and human identity) and nature-conservation-related and climate-protection-related beliefs and value orientations. In doing so, we wanted to shed light on possible underlying conflicts of interest and value-based conflicts, because these conflicts, which are to be assessed as more or less negotiable, would represent a major problem for the acceptance of political measures of climate protection, as well as for the acceptance of nature conservation concerns. First, the results showed that there were substantial similarities between nature conservation beliefs justified by biospheric value orientation (protecting biodiversity) and values and norms relevant for climate protection: There were strong intercorrelations between personal norm, biospheric values, and conservation beliefs that are associated with protecting biodiversity. For conservation beliefs justified by altruistic or egoistic value orientation (preservation of recreational value), the correlations with personal norm and biospheric values were still significant but notably lower. A similar picture emerged for correlations with the acceptance of the four climate protection measures we considered. Again, there were significant positive correlations between personal norm, biospheric values, and biodiversity-related nature conservation beliefs, whereas the correlations between recreational nature conservation beliefs were negatively related or not related at all to the acceptance of the four climate protection measures. In conclusion, there seem to be no substantial value-based conflicts between nature conservation aimed at preserving biodiversity and protecting the climate. In order to increase the acceptance of climate-protection measures, the rather anthropocentric argument that we should preserve the recreational value of nature can be interpreted as a hint toward a conflict of interest. The importance of the expansion of renewable energies and climate protection for the preservation of people’s livelihoods could be emphasized more.

Second, there was a significant correlation between political orientation and the acceptance of all four climate protection measures (the more left-oriented a person’s political orientation, the higher their acceptance). The same was true for the correlations between global human identity and the acceptance measures, but these correlations were even larger. Furthermore, political orientation and global human identity showed medium to large positive correlations with biospheric values and personal norm. Interestingly, the correlations between political orientation and the expressions of nature conservation beliefs were more nuanced. With respect to nature conservation with the goal of preserving biodiversity, there was a slight positive correlation, whereas the correlation with nature protection for recreational value was slightly negative. As mentioned above, recreation-oriented conservation beliefs seem to be associated with lower acceptance of climate protection measures. Furthermore, political orientation seems to be important for nature conservation beliefs (the more right-oriented a person’s political orientation, the higher their recreation-oriented conservation beliefs). This can be seen as a potential conflict between political orientation and nature conservation beliefs, but this is not the case for biodiversity-oriented nature conservation beliefs. For global human identity, there were significant correlations with both conservation beliefs, but the correlation with the recreation-related conservation belief was larger (a belief that has an anthropocentric rationale), which seems logical since global human identity is about identification with humanity and not with nature.

Third, taking into account the possible interrelationships between political orientation and both nature-conservation-related and climate-protection-related beliefs and value orientations with respect to the acceptance of climate protection measures in more detail, we tested structural equation models for each of the four climate protection measures. For all of the four measures, personal norm mediated the relationships between biospheric values and acceptance of the climate protection measures. Likewise, personal norm also mediated the influence of political orientation on the acceptance of the four measures. If global human identity was included as a predictor instead of political orientation, personal norm also mediated the associations with the acceptance of all four measures. These findings support the key role of personal norm in this context.

The results were particularly interesting with regard to two measures, namely, the acceptance of wind turbines and building with wood. Whereas biodiversity-related nature conservation beliefs mediated the influence of biosphere values on the acceptance of photovoltaic systems in cities as well as free-field photovoltaic systems, recreation-related nature conservation beliefs mediated this relationship for the acceptance of wind turbines, but biodiversity-related nature conservation beliefs were not relevant. As already stated above, the aspect of recreation seems to play a specific role when it comes to the acceptance of wind turbines.

The mediation analyses in which building with wood was a criterion showed no mediating effects of either of the nature conservation beliefs. This result could be interpreted as an indication that the respondents might not have reflected on the consequences of this measure for future forest management and use.

## 5. Limitations and further research

The findings presented and discussed here are to be considered embedded in their local, socio-political context. Our sample was representative of the German population. This is certainly a limitation of the study, as Germany is a very densely populated country, which poses a particular challenge for measures with an impact on land use. However, we assume that the main findings reported here might be of relevance to large parts of Europe and also to the US. With regard to the discussion of possible risks of the instrumentalization of conservation narratives by political groups, caution is certainly required; here, for example, German conservation history and its historical instrumentalization by right-wing nationalists play a special role.

The study at hand does not provide the testing of a full-fledged model and does therefore not claim to fully explain the socio-political acceptance of climate protection measures. Instead, we believe that the results might provide indications for a constructive management of climate protection measures and nature conservation. Even though the study focuses specifically on potential conflicts, there are still some limitations.

Regarding the measurement instruments we used, only biospheric values were collected. Not taking all value orientations into account represents a clear limitation of the study presented here. Since the major risk of conflict is expected to arise from a contradiction between biospheric values and climate protection motives, we nevertheless believe that the present findings can make a valuable contribution to the further analysis in this domain and recommend that future studies also include egoistic and altruistic values. Furthermore, we used single items to measure nature conservation beliefs due to the insufficient psychometric properties of the original nine items. Sufficient scales should be developed for future research. It must be mentioned that the correlation-based analyses do not allow causal conclusions. Experimental research designs would be needed to analyze the causal relationships more systematically. Mediation models should be tested with longitudinal data or experimental interventions as they model causal mechanisms. Thus, the results from the mediation analyses in this study should be interpreted as preliminary evidence against a potential intrapersonal conflict between climate protection orientation and nature conservation orientation grounded in biospheric values. Further research based on experimental designs could continue to shed light on potential conflicts by systematically varying the specific impacts and constraints of climate protection measures on nature and biodiversity.

We see a need for further research, especially regarding a more differentiated analysis of political orientation. In the present study, important insights were gained, also regarding possible ties with the concept of global human identity. In fact, this relationship does not seem to be particularly close, as global human identity seems to be linked more to personal ecological norms than to political orientation.

At the same time, it is evident that it is worthwhile to use more differentiated instruments to measure political orientation in future studies to better understand the interplay between political orientation, nature conservation beliefs, and the acceptance of climate protection measures. To further test the findings obtained here, specific populations, such as members of nature conservation associations or right-wing supporters, could be analyzed in more detail in the future.

Recent policy papers and scientific papers have shown a trend toward new topics and technologies such as Carbon Capture (Carbon Capture and Storage, CCS; Carbon Capture and Use, CCU; Cioenergy with Carbon Capture and Storage, BECCS; Direct Air Carbon Capture and Storage, DACCS), use of hydrogen, and Negative Emission Technologies (NET) (Antonini et al., 2020; Borchers et al., 2022). Possible conflicts associated with these upcoming technologies and associated impacts on nature and landscapes may act as drivers – or as barriers – for transformation processes in our society. We hope that our study can inspire further, more differentiated analyses in this field.

## Data availability statement

The raw data supporting the conclusions of this article will be made available by the authors, without undue reservation.

## Ethics statement

Ethical review and approval was not required for the study on human participants in accordance with the local legislation and institutional requirements. The patients/participants provided their written informed consent to participate in this study.

## Author contributions

AB, EM, MB, and KB contributed to conception and design of the study. AB and LE contributed to the analysis strategies and interpretation of data and wrote the first draft of the manuscript. KB wrote sections of the manuscript. LE organized the database and performed the statistical analysis. EM supervised the project. All authors provided critical feedback and helped shape the manuscript.

## Funding

The study was conducted in the frame of the interdisciplinary research initiative “SmartProSys: Intelligent Process Systems for the Sustainable Production of Chemicals.” The research was funded by the Federal State of Saxony-Anhalt, Ministry for Science, Energy, Climate Protection and the Environment. We acknowledge support by the Open Access Publication Fund of Magdeburg University.

## Conflict of interest

The authors declare that the research was conducted in the absence of any commercial or financial relationships that could be construed as potential conflicts of interest.

## Publisher’s note

All claims expressed in this article are solely those of the authors and do not necessarily represent those of their affiliated organizations, or those of the publisher, the editors and the reviewers. Any product that may be evaluated in this article, or claim that may be made by its manufacturer, is not guaranteed or endorsed by the publisher.

## Footnotes

^1^According to a representative survey of *N* = 2,044 people conducted in 2019 by the German Federal Agency for Nature Conservation, only 8% of respondents reject the extension of renewables in principle (BfN (Bundesamt für Naturschutz), 2022)).

^2^The participants received a financial compensation.

^3^Stern et al. (1998) decided for a simplified 7-point scale because their interviews were conducted *via* telephone.

^4^For a CFA model that combined PN and BV, used MLR estimation, and allowed the two latent factors to correlate (*r* = 0.64), the fit indices indicated good to excellent model fit (robust CFI = 0.992, robust RMSEA = 0.064, SRMR = 0.024; Hu and Bentler, 1999).

^5^Initially, we included nine items in the survey to measure NCB. After extensive analyses of possible factor structures underlying these items and excluding some items, no model showed sufficient psychometric properties. Furthermore, the hypothesized factor structure yielded computational problems, indicating an inadequate item-factor configuration. Thus, we decided to use two items as “marker variables” that represented the two beliefs best in our eyes.

^6^We conducted one-sample t-tests with a right-tailed alternative hypothesis (*μ* > *μ_0_*).

^7^The significance of the difference between the correlations was tested with Pearson and Filon’s *z* (Pearson and Filon, 1898; Raghunathan et al., 1996).
