# Supplementary material for: Nature conservation versus climate protection: a basic conflict of goals regarding the acceptance of climate protection measures?
Source: Front Psychol. 2023 Jun 26;14:1114677. doi: 10.3389/fpsyg.2023.1114677 (PMC10330816; doi:10.3389/fpsyg.2023.1114677)
Supplement: Supplementary file 1 [file Data_Sheet_1.docx]

**Table A1**

*Descriptive Statistics for Items (imputed data set).*

|  |  | *Min.* | *Max.* | *Median* | *Mean* | *SD* | *Skewness* | *Kurtosis* |
| --- | --- | --- | --- | --- | --- | --- | --- | --- |
| Biosperic Values | |  |  |  |  |  |  |  |
|  | WO01_01 | -1 | 7 | 5 | 5.14 | 1.75 | -0.90 | 0.58 |
|  | WO01_02 | -1 | 7 | 5 | 4.59 | 1.92 | -0.58 | -0.14 |
|  | WO01_03 | -1 | 7 | 6 | 5.38 | 1.68 | -1.04 | 0.76 |
| Personal Norm | |  |  |  |  |  |  |  |
|  | PN01_01 | 1 | 5 | 4 | 3.49 | 1.18 | -0.58 | -0.40 |
|  | PN01_02 | 1 | 5.18 | 4 | 3.54 | 1.14 | -0.59 | -0.25 |
|  | PN01_03 | 0.71 | 5.69 | 3.50 | 3.41 | 1.17 | -0.45 | -0.48 |
| Global Human Identity | |  |  |  |  |  |  |  |
|  | GH01_01 | 1 | 7.55 | 4 | 4.32 | 1.70 | -0.22 | -0.66 |
|  | GH01_02 | 0.76 | 7.48 | 5 | 4.59 | 1.73 | -0.42 | -0.57 |
|  | GH01_03 | 1 | 7.59 | 5 | 4.93 | 1.56 | -0.66 | 0.12 |
|  | GH01_04 | 1 | 8.34 | 5 | 4.58 | 1.63 | -0.40 | -0.36 |

*Note. N = 1,427*

**Table A2**

*Descriptive Statistics for Scale Score (based on mean factoring).*

|  | *Min.* | *Max.* | *Median* | *Mean (t)* | *SD* | *Skewness* | *Kurtosis* |
| --- | --- | --- | --- | --- | --- | --- | --- |
| Personal Norm (*n* = 1,427) | 0.90 | 5.12 | 3.67 | 3.48 (16.70^***^) | 1.09 | -0.57 | -0.18 |
|  |  |  |  |  |  |  |  |
| Biosperic Values (*n* = 1,427) | -1 | 7 | 5.33 | 5.03 (48.31^***^) | 1.59 | -0.78 | 0.43 |
|  |  |  |  |  |  |  |  |
| Nature conservation beliefs |  |  |  |  |  |  |  |
| NCB (recreational) (*n* = 1,427) | 1 | 5 | 3 | 2.98 (-0.77) | 1.13 | 0.004 | -0.67 |
| NCB (biodiversity) (*n* = 1,427) | 1 | 5 | 4 | 4.10 (48.81^***^) | 0.85 | -0.68 | 0.13 |
|  |  |  |  |  |  |  |  |
| Global Human Identity (*n* = 1,427) | 1 | 7.74 | 4.65 | 4.61 (15.29^***^) | 1.50 | -0.37 | -0.28 |
|  |  |  |  |  |  |  |  |
| Political Orientation (*n* = 1,297) | 1 | 10 | 6 | 5.93 (8.90^***^) | 1.76 | -0.03 | 0.47 |
|  |  |  |  |  |  |  |  |
| Acceptance of Climate Protection Measures |  |  |  |  |  |  |  |
| Wind Turbines (*n* = 1,280) | 1 | 5 | 5 | 4.19 (41.16^***^) | 1.03 | -1.20 | 0.83 |
| Photovoltaic (city) (*n* = 1,219) | 1 | 5 | 5 | 4.55 (71.96^***^) | 0.75 | -1.67 | 2.37 |
| Photovoltaic (free-field) (*n* = 1,042) | 1 | 5 | 5 | 4.18 (35.48^***^) | 1.07 | -1.29 | 0.99 |
| Building with wood (*n* = 1,093) | 1 | 5 | 4 | 3.91 (29.02^***^) | 1.04 | -0.68 | -0.12 |

*Note. t = t-value for a one-sample t-test with right-tailed alternative hypothesis (sample mean > Likert-scale midpoint). ^***^ p < .001.*

**Table A3**

*Test statistics for univariate and multivariate normality tests for personal norm.*

|  |  | W (W') | | | |
| --- | --- | --- | --- | --- | --- |
| Item level | |  | | | |
| PN01_01 | | .89 | | | |
| PN01_02 | | .89 | | | |
| PN01_03 | | .90 | | | |
|  | | W (W') | Henze-Zirkler | Mardia Skewness | Mardia Kurtosis |
| Scale level | |  |  |  |  |
| Personal Norm | | .94 | 74.71 | 171.51 | 24.42 |

Note. N = 1,427. W = Shapiro-Wilk test statistic (perfect normality = 1.00; all test statistics were the same when the Shapiro-Francia test was applied). All ps < .001.

**Table A4**

*Test statistics for univariate and multivariate normality tests for the biospheric value orientation.*

|  |  | W | | | |
| --- | --- | --- | --- | --- | --- |
| Item level | |  | | | |
| WO01_01 | | .88 | | | |
| WO01_02 | | .92 | | | |
| WO01_03 | | .86 | | | |
|  | | W | Henze-Zirkler | Mardia Skewness | Mardia Kurtosis |
| Scale level | |  |  |  |  |
| Biopsheric Value Orientation | | .93 | 45.72 | 693.43 | 31.09 |

Note. N = 1,427. W = Shapiro-Wilk test statistic (perfect normality = 1.00; all test statistics were the same when the Shapiro-Francia test was applied). All ps < .001.

**Table A5**

*Test statistics for univariate and multivariate normality tests for global human identity.*

|  |  | W | | | |
| --- | --- | --- | --- | --- | --- |
| Item level | |  | | | |
| GH01_01 | | .94 | | | |
| GH01_02 | | .93 | | | |
| GH01_03 | | .91 | | | |
| GH01_04 | | .94 | | | |
|  | | W | Henze-Zirkler | Mardia Skewness | Mardia Kurtosis |
| Scale level | |  |  |  |  |
| Global Human Identity | | .97 | 26.59 | 548.31 | 35.86 |

Note. N = 1,427. W = Shapiro-Wilk test statistic (perfect normality = 1.00; all test statistics were the same when the Shapiro-Francia test was applied). All ps < .001.

**Table A6**

*Test statistics for univariate normality tests for single-item measures.*

|  |  | W |
| --- | --- | --- |
| Item level |  |  |
| Nature conservation beliefs | |  |
|  | NCB (recreational) (*n* = 1,427) | .92 |
|  | NCB (biodiversity) (*n* = 1,427) | .83 |
| Political Orientation (*n* = 1,297) | | .96 |
| Acceptance of Climate Protection Measures | |  |
|  | Wind Turbines (*n* = 1,280) | .76 |
|  | Photovoltaic (city) (*n* = 1,219) | .64 |
|  | Photovoltaic (free-field) (*n* = 1,042) | .75 |
|  | Building with wood (*n* = 1,093) | .85 |

Note. W = Shapiro-Wilk test statistic (perfect normality = 1.00; all test statistics were the same when the Shapiro-Francia test was applied). All ps < .001.
