# Supplementary figures and images for: Nature conservation versus climate protection: a basic conflict of goals regarding the acceptance of climate protection measures?
Source: Front Psychol. 2023 Jun 26;14:1114677. doi: 10.3389/fpsyg.2023.1114677 (PMC10330816; doi:10.3389/fpsyg.2023.1114677)

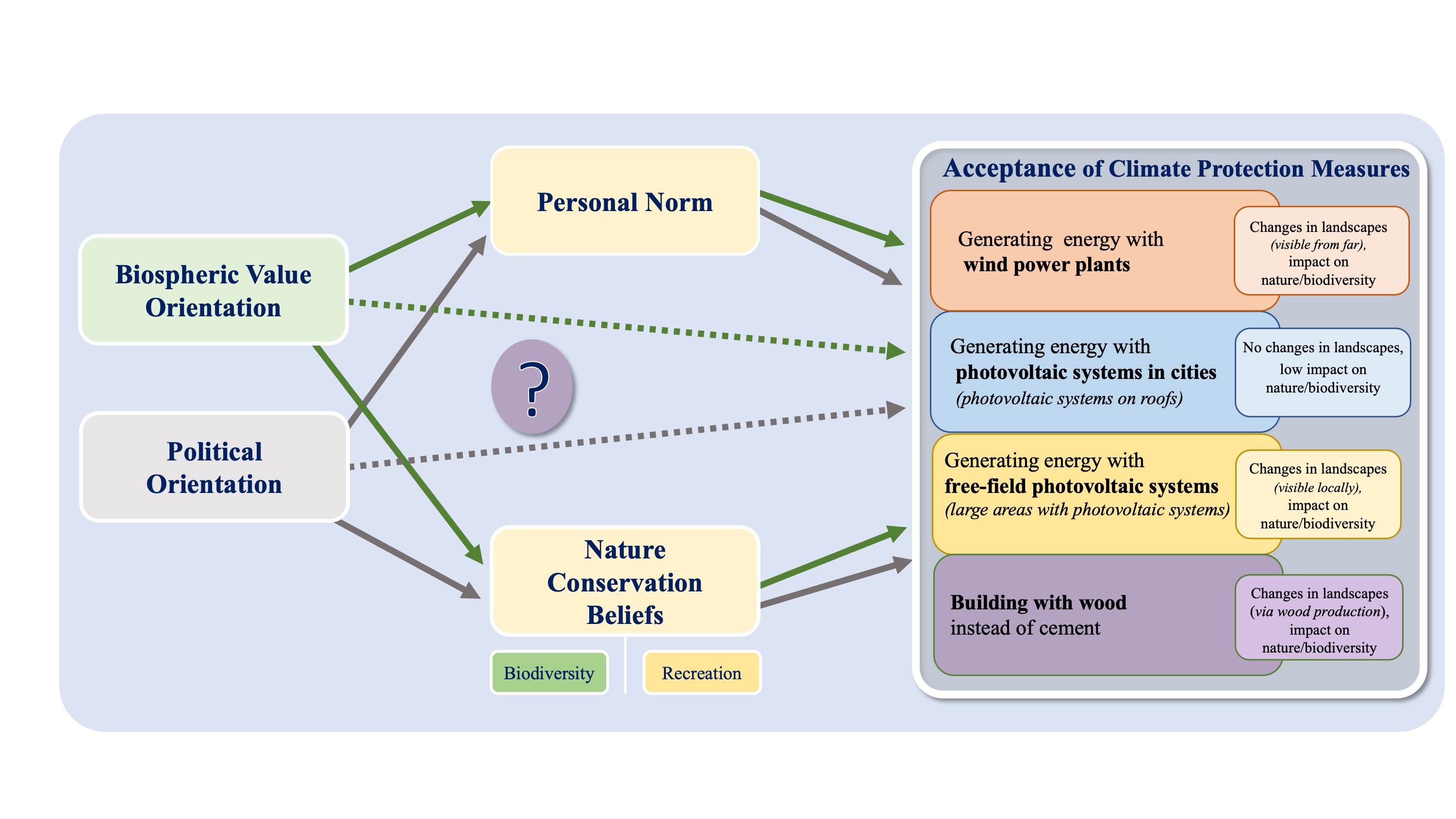

Supplement: Supplementary file 2 [file Image_1.JPEG]
